# Towards a “4I” approach to personalized healthcare

**DOI:** 10.1186/2001-1326-1-14

**Published:** 2012-07-30

**Authors:** Philip R O Payne, Clay B Marsh

**Affiliations:** 1 The Ohio State University Wexner Medical Center, Department of Biomedical Informatics, 3190 Graves Hall, 333 West 10th Avenue, Columbus, OH, 43210, USA; 2 The Ohio State University Wexner Medical Center, Department of Internal Medicine and Center for Personalized Healthcare, 260 Meiling Hall, 370 West 9th Ave, Columbus, OH, 43210; 3 Center for Personalized Healthcare, The Ohio State University Wexner Medical Center, Columbus, OH, USA

**Keywords:** Individualized Medicine, Informatics, Organization & Administration

## Abstract

Personalized healthcare holds the promise of ensuring that every patient receives optimal wellness promotion and clinical care based upon his or her unique and multi-factorial phenotype, informed by the most up-to-date and contextually relevant science. However, achieving this vision requires the management, analysis, and delivery of complex data, information, and knowledge. While there are well-established frameworks that serve to inform the pursuit of basic science, clinical, and translational research in support of the operationalization of the personalized healthcare paradigm, equivalent constructs that may enable biomedical informatics innovation and practice aligned with such objectives are noticeably sparse. In response to this gap in knowledge, we propose such a framework for the advancement of biomedical informatics in order to address the fundamental information needs of the personalized healthcare domain. This framework, which we refer to as a “4I” approach, emphasizes the pursuit of research and practice that is information-centric, integrative, interactive, and innovative.

## Background

The objective of personalized healthcare is to ensure that each patient has the best clinical outcome by tailoring both preventative measures and treatments to meet their unique needs and characteristics. Achieving such a vision requires not only the collection and application of the best possible data, information, and knowledge during each patient encounter, but also, learning from each encounter and engaging patients and their families in the healthcare process. This vision of a learning health care system can improve the outcomes and quality of care for the individual patient, their family, and their community.

An innovative and paradigm-shifting approach to conceptualizing personalized healthcare has been described by Weston and Hood using the moniker of “P4 Medicine” - where it was proposed that our fundamental approach to disease prevention, diagnosis, and treatment must transition from being a primarily reactive model to one that is predictive, personalized, preventive and participatory [1]. In this model, it is envisioned that our fundamental approach to the delivery of healthcare will be shifted from an emphasis on treating illness to the early and continuous prevention of disease and the promotion of wellness. Furthermore, under this paradigm, the patient becomes an integral part of the healthcare delivery ecosystem, taking an active role in the identification and modification of disease related risk factors, while also assuming responsibility for critical aspects of their ongoing care (moving from being a passive consumer of clinical care to an active member of the overall healthcare team). Unfortunately, it is widely noted that the current healthcare delivery workflows (including essential data, information, and knowledge management methods) are not well aligned with the P4 paradigm, thus impeding the implementation of the model [2-6].

## A primer on biomedical informatics and its role in supporting “P4 medicine”

The scientific discipline of Biomedical Informatics emerged over the last several decades as a catalyst for the discovery, study, and delivery of innovative solutions to data, information, and knowledge management needs in the biomedical and healthcare domains. Biomedical Informaticians use domain-specific theories and methods to interpret and reason upon complex data in order to deliver contextually appropriate information and knowledge at multiple end-points, such as the laboratory, point-of-care, or community settings. As can be readily ascertained, this continuum of data, information, and knowledge management is central to the premises underlying P4 medicine. Unfortunately, current approaches to basic science research, clinical care, and biomedical informatics are often poorly integrated, yielding clinical decision-making processes that do not take advantage of up-to-date scientific knowledge [2,3,7]. There are an increasing number of systems modelling and *in-silico* knowledge synthesis techniques that can provide investigators with the tools to address such information needs, but their adoption and evaluation remains an area of early and open research [4-8]. Given increasing concerns over barriers to translating discoveries from the laboratory to the clinic or community, such high-throughput informatics methods are very desirable, and in our opinion, central to the P4 paradigm [4,7-10]. We believe that the fundamental barrier preventing such rapid and systematic translation between research and clinical care is lack of unification between data generation, as regularly occurs in the laboratory, clinical, and community settings; and knowledge generation, which is the fundamental pursuit of research. This lack of unification is attributable to a number of factors, including socio-technical and regulatory barriers, as well as a lack of sufficiently robust and widely adopted informatics platforms intended to “shorten the distance” between data and knowledge generation [4,5].

## The “4I” Approach

**Figure 1:**
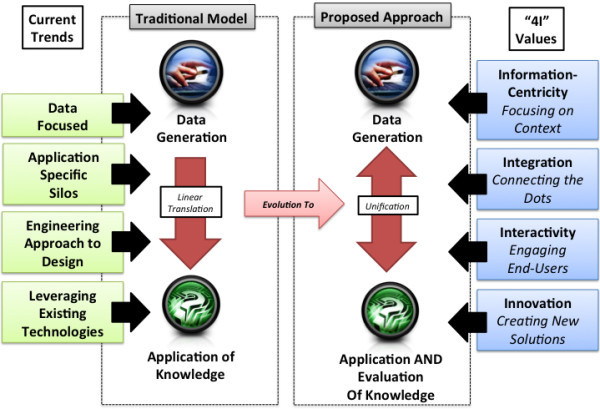
**Conceptual model for the "4I” approach to P4 Medicine.** In this model, the traditional separation of clinical care and research employing a linear approach to knowledge translation evolves to support the unification of data and knowledge generation. This model involves the shifting of Biomedical Informatics foci from current trends involving solution that are: 1) data focused; 2) specific to application areas (e.g., silos aligned with basic and clinical research or clinical care); 3) implemented using an engineering approach to the user experience which values function over form; and 4) that regularly leverages existing technologies; to a new paradigm supported by four core values: 1) information-centricity; 2) integration; 3) interactivity, and 4) innovation.

Building upon the P4 Medicine model and the preceding gaps in the state of Biomedical Informatics practice, we believe that a corresponding “4I” approach to Biomedical Informatics at the interface of research, education, and clinical care is needed. At a high level, this approach is intended to achieve unrealized benefits in terms of unifying data and knowledge generation as introduced earlier. Our framework (Figure 1) seeks to describe a core set of values that guides basic and applied Biomedical Informatics science to ensure the ongoing implementation and support of P4 medicine:

### **Information-centric**

Our focus is not simply collecting and transacting data, but also providing appropriate context to such data to transform it into mission-critical knowledge. As the range of data types needed to deliver personalized healthcare expands in complexity and size, such an information-centric approach will require systems-level approaches to data, information, and ultimately knowledge management that extend well beyond singular information systems. Such a systems-level approach is needed to create an “information fabric” that supports novel research, the training of healthcare and life science professionals, and the delivery of care both within and beyond organizational boundaries.

### **Integrative**

Building on the concept of information-centricity, it is imperative that we begin to focus on approaches to “connecting the dots” between heterogeneous information and knowledge types in a scalable and high-throughput manner. For example, we have large amounts of clinical data that are collected in electronic health records, and similarly, large amounts of genomic data that is generated via advanced laboratory instruments. However, our ability to understand the linkages between such data types and knowledge generation in order to improve clinical care and disease prevention is often limited to the intuition of individual investigators. At the same time, advances in artificial intelligence and knowledge-based system design as applied to biomedicine can allow computational agents to reason across such data types and simultaneously mine the available literature and other knowledge resources. As an outcome of this activity, a large number of testable hypotheses that can inform important clinical evidence in support of personalized healthcare can be generated and tested.

### **Interactive**

While computers have immense capabilities of collecting, storing, and analyzing large amounts of data and information, their ability to identify and assess important patterns in these datasets is limited. In comparison, humans have unique and presently non-reproducible cognitive strengths in the area of pattern recognition and high-level problem solving. As a result, any approach to leveraging Biomedical Informatics in the context of a complex environment presented by the practice of personalized healthcare, must involve an optimal combination of both human and computational workflows. Unfortunately, the area of human-computer interaction in the biomedical domain remains immature, with a primary focus being placed more on technical solutions to informatics needs, rather than the user experience. Therefore, we need to better develop and apply such approaches in order to improve the ability of researchers, educators, clinicians, patients, and their families to use advanced information technology platforms informed by advances in Biomedical Informatics.

### **Innovative**

Finally, and perhaps most importantly, we must continue to “push the envelope” of Biomedical Informatics innovation in order to create new solutions to the problems constantly emerging throughout the complex biomedical and healthcare environment. The history of Biomedical Informatics clearly illustrates that almost every innovative solution to a fundamental data, information, or knowledge management problem in the biomedical and healthcare domains leads to more questions and possibilities for advancing the field. Therefore, we must not be satisfied with the theories, methods, and tools we currently have in our Biomedical Informatics “tool box”, but instead, should constantly seek new avenues of innovation. Doing so will also require the creation of partnerships and funding models capable of supporting such innovation, particularly given the nearly tectonic shifts occurring in our national research enterprise.

## Discussion

In order to further contextualize and illustrate the potential benefits of the proposed “4I” approach to personalized healthcare, in the discussion below, we provide a exemplary P4-focused use case, and then compare and contrast Biomedical Informatics approaches to addressing that use case’s information needs and implemented based upon:

Contemporary trends in Biomedical Informatics practice that tend to emphasize the implementation of solutions that are: 1) data focused; 2) specific to application areas (e.g., silos aligned with basic and clinical research or clinical care); 3) designed using an engineering approach to the user experience which values function over form; and 4) leverage existing technologies; and

The principles and values that underlie the proposed “4I” approach, representing an ideal future state for such data, information, and knowledge management solutions.

### Use case

A large academic healthcare center (AHC) seeks to pilot the targeted sequencing of patient genomes in order to inform pharmacogenomic decision support at the point of care. As part of this project, the AHC intends to inform such decision support rules using knowledge generated via current basic science investigations, and then study the impact of such interventions on patient outcomes and quality/safety of care.

### Contemporary biomedical informatics solutions addressing this use case

Patient samples will be sequenced, and the resulting data sets processed using available analytical tools informed by the current pharmacogenomics knowledge base in order to identify clinically relevant markers in each individual’s genomes. Such synthesized results are reported in an aggregate format, indicating the patients risk’s of adverse drug reactions and/or the potential usefulness of genetically targeted therapies (if applicable). Given the limitations of common, commercial EHR platforms, this report is stored as a PDF file and attached to the individual patients electronic medical records. During clinical encounters, providers are alerted to the presence of this report and encouraged to use it when considering therapeutic options for the patient. Subsequently, a combination of data analytics targeting discrete variables of interest as well as the semi-automated review of clinical notes, is conducted for patients involved in the pilot, in order to determine what impacts the availability of the aforementioned genomic data had on patient outcomes and quality/safety of care in a retrospective manner.

### “4I”-Based biomedical informatics solutions addressing this use case

Patient samples will be sequenced, and the resulting raw data stored in a distributed data repository that is appropriately tuned for the storage and retrieval of “big data.” During a given patient encounter, should a medication order be entered, a real time process will: 1) determine if genomic data is available for the patient; 2) query a dynamic knowledge base consisting of expertly curated rules as well as rules extracted through the periodic processing and analysis of available bibliographic data sets, in order to identify potential correlations between the medication being considered by the clinician and available and current pharmacogenomc knowledge; 3) apply those rules to the patient’s unique genomic and phenotypic variables; and 4) generate a point-of-care alert with recommendations based on the preceding analysis. If the this process is invoked as described, the patient’s records would also be flagged, and a regularly occurring analytical “agent” would analyze near, medium, and long term patient outcomes and quality/safety of care indicators, and present such analyses in a dashboard format available to patients, clinicians, and researchers involved in the project. Finally, all of the aforementioned measures would be designed and implemented based on exhaustive end-user focused human factors and workflow analyses, intended to optimize workflow integration and user experience for all stakeholders.

## Conclusion

We believe that the preceding vision of P4 Medicine and the “4I” approach is both essential and critical to advance the state of healthcare in a manner that increases quality and safety while reducing costs and increasing patient access. However achieving this vision will require major changes in our approach to data, information, and knowledge management, particularly with regard to unifying such activities. As such, we argue that a “4I” approach to address the foundational information needs of P4 Medicine, based upon the core values of information centricity, integration, interactivity, and innovation, is of the utmost importance. In presenting these views, we hope to catalyze a vigorous dialogue concerning the development of a Biomedical Informatics research and development roadmap that is closely aligned with that of the P4 Medicine paradigm.

## Abbreviations

P4, Predictive Personalized, Preventive and Participatory; 4I, Information centricity Integration, Interactivity, and Innovation.

## Competing interests

The authors have no financial or non-financial competing interests relevant to the content of this commentary.

## Authors contributions

PP and CM were responsible for the conceptualization, development, and final approval of this commentary.

## Authors information

***Philip R.O. Payne, PhD:*** Dr. Payne has been a faculty member in the Department of Biomedical Informatics at The Ohio State University since 2006, and is an internationally recognized leader in the fields of clinical research informatics and translational bioinformatics. He currently serves as Chair of the Department of Biomedical Informatics within The Ohio State University College of Medicine, and as Executive Director of The Ohio State University Wexner Medical Center’s Informatics Research and Development unit. He also serves in leadership roles for the Biomedical Informatics Programs/Cores affiliated with both The Ohio State University Center for Clinical and Translational Science and The Ohio State University Comprehensive Cancer Center. Dr. Payne’s research portfolio is actively supported by a combination of NCATS, NLM, and NCI awards and contracts and spans a spectrum from the development of core clinical and translational research data management platforms to the study of workflow and human factors that may serve to influence the optimal use of Health Information Technology. Dr. Payne received his Ph.D. with distinction in Biomedical Informatics from Columbia University. Prior to pursuing graduate training in Biomedical Informatics, Dr. Payne served in a number of technical and leadership roles at both the UCSD Shiley Eye Center and UCSD Moores Cancer Center. Dr. Payne’s leadership in the clinical and translational research informatics communities has been recognized through his appointment to numerous national steering and advisory committees as part of the American Medical Informatics Association (AMIA), Association for Computing Machinery (ACM), National Cancer Institute caBIG initiative, and the CTSA consortium, as well as his engagement as a consultant to academic health centers throughout the United States and the Institute of Medicine. Dr. Payne is the author of over 100 publications focusing on the intersection of biomedical informatics and the clinical and translational science domains, including several seminal reports that have served to define a new sub-domain of biomedical informatics theory and practice specifically focusing upon clinical research applications.

***Clay B. Marsh, MD:*** Dr. Marsh is the Vice Dean of Research of The Ohio State University College of Medicine. He also serves in a number of leadership roles throughout The Ohio State University Wexner Medical Center, including his appointments as Senior Associate Vice President for Research in the Office of Health Sciences, Director of the Center for Critical Care and Respiratory Medicine, and Executive Director, Center for Personalized Healthcare. Dr. Marsh has been a member of the medical staff at Ohio State since 1985. He earned his medical degree from West Virginia University and completed his residency in internal medicine at Ohio State, where he served as chief resident. Dr. Marsh has won numerous teaching awards and has been recognized nationally for his research and devotion to teaching and mentorship of medical students, residents and fellows. He has published more than 230 journal articles, abstracts and book chapters, and holds one patent with five more pending. He is associate editor of the Journal of Investigative Medicine and the American Journal of Physiology. He also has served as chairman of the board of the Stanley Sarnoff Research Foundation and as a national leader in pulmonary medicine on the Battelle Bioinitiative in Pulmonary Medicine.
